# Effect of negative emotions evoked by light, noise and taste on trigeminal thermal sensitivity

**DOI:** 10.1186/1129-2377-15-71

**Published:** 2014-11-07

**Authors:** Guangju Yang, Lene Baad-Hansen, Kelun Wang, Qiu-Fei Xie, Peter Svensson

**Affiliations:** 1 Department of Prosthodontics and Center for Oral Functional Diagnosis, Treatment and Research, Peking University School and hospital of Stomatology, Zhongguancun Nandajie 22, 100081 Beijing, China; 2 Section of Clinical Oral Physiology, Department of Dentistry, Aarhus University, Aarhus, Denmark; 3 Center for Sensory-Motor Interaction (SMI), Department of Health Science and Technology, Aalborg University, Aalborg, Denmark; 4 Department of Dental Medicine, Karolinska Institute, Huddinge, Sweden

**Keywords:** Quantitative sensory testing, Affective state, Thermal sensitivity, Light, Noise, Taste

## Abstract

**Background:**

Patients with migraine often have impaired somatosensory function and experience headache attacks triggered by exogenous stimulus, such as light, sound or taste. This study aimed to assess the influence of three controlled conditioning stimuli (visual, auditory and gustatory stimuli and combined stimuli) on affective state and thermal sensitivity in healthy human participants.

**Methods:**

All participants attended four experimental sessions with visual, auditory and gustatory conditioning stimuli and combination of all stimuli, in a randomized sequence. In each session, the somatosensory sensitivity was tested in the perioral region with use of thermal stimuli with and without the conditioning stimuli. Positive and Negative Affect States (PANAS) were assessed before and after the tests. Subject based ratings of the conditioning and test stimuli in addition to skin temperature and heart rate as indicators of arousal responses were collected in real time during the tests.

**Results:**

The three conditioning stimuli all induced significant increases in negative PANAS scores (paired *t*-test, *P* ≤0.016). Compared with baseline, the increases were in a near dose-dependent manner during visual and auditory conditioning stimulation. No significant effects of any single conditioning stimuli were observed on trigeminal thermal sensitivity (*P* ≥0.051) or arousal parameters (*P* ≥0.057). The effects of combined conditioning stimuli on subjective ratings (*P* ≤0.038) and negative affect (*P* = 0.011) were stronger than those of single stimuli.

**Conclusions:**

All three conditioning stimuli provided a simple way to evoke a negative affective state without physical arousal or influence on trigeminal thermal sensitivity. Multisensory conditioning had stronger effects but also failed to modulate thermal sensitivity, suggesting that so-called exogenous trigger stimuli e.g. bright light, noise, unpleasant taste in patients with migraine may require a predisposed or sensitized nervous system.

## Background

Migraine is an inherited, episodic disorder involving changes in responsivity of the sensory systems. For example, migraine patients complain of headache attacks with increased sensitivity to e.g. light or sound. Some patients mention that otherwise pleasant odors are unpleasant during the attacks. The disorder is considered as a disturbance in the brain of the subcortical aminergic sensory modulatory systems [1]. Overall, these observations could indicate that the migrainous brain does not habituate to signals in a normal way [2-4].

For a biological system, perception often requires information emanating from sensors of multiple modalities [5]. For instance, human perception of food flavor and texture during consumption involves taste, intraoral somatosensory function (thermal and mechanical), vision, olfaction, and auditory signals that contribute to the overall appreciation of food. Different sensory modalities may interact in a non-linear way as multi-modal sensory integration (MSI) [6]. Moreover, observations that the somatosensory system may modulate activity in auditory brain regions become clinically relevant [7]. The perception of migraine headache is uniquely exacerbated during exposure to ambient light as compared with the pain level perceived in the dark [8].

There are several theories to explain MSI. One of them is the motivational priming model, which suggested the affective state as a mediator [9]. The pain sensitivity may be enhanced by evoked unpleasant affective states, while attenuated by pleasant affective states and high arousal augments both effects [9]. Visual stimuli used in previous studies examining emotional processes have been either Pictures of Facial Affect or the International Affective Picture System (IAPS) [10,11]. Music may also evoke comparable emotional responses across different musical categories [11,12]. However, the individual background, such as culture and education, is important for the results of such tests [13]. Therefore, for example bright light and a chainsaw noise, to which responses are less ethnic, cultural, or education-dependent, could be useful in some studies. Sucrose has been reported to reduce pain in children, while the effect of unpleasant bitter gustatory stimulation on pain has not been systematically studied [14,15].

Another possible effect of cross-modality sensory regulation could be simply a distraction effect reducing the perceived stimulus intensity by diverting attention away from pain processing [16-18]. However, one study indicated that distraction tasks that demand greater attention processing do not produce greater reductions in pain [19].

It is obvious that real life experiences mostly rely on the presence of combined stimuli coming from different modalities. For example, music is often used to enhance the emotion impact of movies. The enhancing effect of combined presentation of different modalities is, however, intuitive and understudied [11].

Quantitative sensory testing (QST) provides a reliable and accurate tool for examination of somatosensory function [20,21]. One study, which evaluated the effect of emotion on sensory-discriminative parameters, indicated that changes in pain sensitivity are more often observed for pain threshold measures than for pain tolerance measures [22].

The present study aimed to assess the effect of, and possible interactions among different unpleasant conditioning stimulus modalities, i.e. visual (light), auditory (noise), gustatory (bitter taste) on affective state, physiological arousal and thermal somatosensory sensitivity in the trigeminal system of young healthy human participants. The following hypotheses were tested: a. the thermal sensitivity of the participants is increased during exposure to conditioning stimuli compared with no conditioning stimuli; b. the unpleasant stimuli evoke negative affective states and physiological arousal compared with baseline; c. combination of conditioning stimuli into a multi-sensory conditioning stimulus exerts larger changes in outcome parameters compared with single conditioning stimuli.

## Methods

### Participants

Young healthy participants in this study were recruited from university students through the webpage [23] and advertisements at Aarhus University, Denmark. Inclusion criteria were: age between 20 to 30 years old, physically and mentally healthy. Exclusion criteria were: Optical, auditory, gustatory functional disorders; infection in the tested area (perioral region); ongoing orofacial pain, or reported chronic pain in last 6 months; serious systemic diseases; medicine intake with effect on the central nervous system in the last 2 weeks; former experience with similar experiments. Twenty-five people responded to the advertisements. Finally, 20 participants aged between 20 to 30 years old (26.6 ± 3.2), 10 females and 10 males met the criteria and were included in this study.

The study adhered to the Helsinki declaration II and was approved by the local ethics committee (NO. 1-10-72-31-13) and all participants gave written informed consent.

### Study procedure

**Figure 1:**
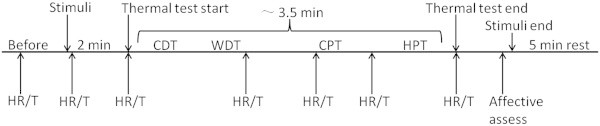
**One thermal somatosensory test consists of: before stimuli (0.5 min), stimulus habituation period (2 min), thermal test (~3.5 min), recovery period (5 min).** ‘HR’ = Heart Rate record, ‘T’ = face skin temperature record. Cold Detection Threshold (CDT), Warmth Detection Threshold (WDT), Cold Pain Threshold (CPT), Heat Pain Threshold (HPT).

The twenty participants each attended four experimental sessions. In each session the participants received one of four conditions: **A.** visual stimulation with bright light alone, **B.** auditory stimulation with recorded chainsaw noise alone, **C.** gustatory stimulation with bitter taste alone, and **D.** multisensory stimulation with gustatory stimulation combined with bright light and chainsaw noise. The four conditions were applied in a randomized sequence produced by Microsoft Excel. There was an interval of about one week between sessions A, B and C. Session D was performed on the same day as session C after a 30 min rest period. A total of six thermal sensory tests were performed over perioral region in each participant in the **A.** visual or **B.** auditory sessions: I baseline 1, II with conditioning stimulus 1; III baseline 2, IV with conditioning stimulus 2; V baseline 3, VI with conditioning stimulus 3. Conditioning stimuli 1–3 (visual, auditory depending on group) were of different intensity (see below). In the **C/D** gustatory and multisensory session, four thermal sensory tests were performed over perioral region in each participant: I baseline 1, II with gustatory conditioning stimulus; III baseline 2, IV with multisensory conditioning stimulus (see below). There was a 2 min adaption period before each test and an interval of 5 min between tests [24,25]. Each session lasted approximately 60 min for each participant (Figure 1 shows the time line of 1 thermal test including the conditioning stimulus).

The tests were performed in a quiet room with temperature at 20–22°C. All participants were required to refrain from ingestion of all food, beverages, and oral care products for a minimum of 2 hour before arrival. The participants were free from head colds and allergies on the test days.

### Conditioning stimuli

#### Visual stimuli

Visual stimuli were created using three levels of bright light shined directly at the participants’ eyes in a randomized sequence produced by Microsoft Excel: (i) 1000 lux bright light; (ii) 3000 lux bright light; (iii) 9000 lux bright light [26,27]. The bright light was generated using dental chair lamp with an area of 14 × 9 cm (ESTETICA E80, KaVo Germany) and calibrated by a Luxmeter (LX1010B+, Malmbergs, Sweden). There was a control test before each test without visual stimuli to eliminate timing effect, in which the participants wore eyeshades and closed their eyes to make sure they did not see any light.

#### Auditory stimuli

Participants were instructed to remain awake with eyes covered by eyeshades and were exposed to recorded sounds of an electric chainsaw at three different intensities (60 dB, 75 dB, 90 dB) in randomized sequence produced by Microsoft Excel [6,28]. The noise was generated by a computer through loud-speakers and measured by Sound Pressure Lever Meter (RadioShack 33–2055, USA). A control test was performed before each test without sound stimulus to eliminate the effect of timing.

#### Gustatory stimulus

Participants were instructed to rinse with distilled water at least four times over a 1 min period prior to testing. Gelatin was the vehicle for gustatory stimuli. The participants were asked to hold 20 ml gelatin (2% bovine skin gelatin, Sigma-Aldrich, St. Louis, USA) in their mouth with either bitter (0.1% quinine, Sigma-Aldrich, adult toxic dose, 2.5 – 4 g) or neutral taste (2% gelatin) [29]. The gustatory stimuli were presented at room temperature (~21**°**C) with an at least four times distilled water rinse after each test for 5 min until the taste has been washed out [30].

#### Multisensory stimuli

The three conditioning stimulus modalities (visual 9000 lux light, auditory 90 dB noise and gustatory 0.1% quinine gelatin) were presented simultaneously to each participant with a control test (no visual, no auditory & neutral taste).All the conditioning stimuli were given in repeated trials, 3 trials for the visual or auditory tests, and 1 trial for the gustatory or combination tests. The stimuli were started 2 min before each thermal test, and ended after the emotion evaluations; thus, each episode of stimulus lasted about 7 min (Figure 1).

### Thermal sensitivity measures

Participants were positioned in a comfortable dental chair. The measures were performed in the right perioral region with the thermode placed on the right angle of the mouth. The thermal tests were performed using a Medoc Pathway with ATS thermode (30 mm × 30 mm, square surface, MEDOC, Israel). Care was taken throughout the session to keep the test sites during the repeated testing the same. Cold and warmth detection thresholds (CDT, WDT) were measured first. Then cold pain and heat pain thresholds (CPT, HPT) were determined [20,21]. The mean threshold temperature of three consecutive measurements was calculated. All thresholds were obtained with ramped stimuli (1°C/s) that were terminated when the participants pressed a button. For thermal detection thresholds the ramp back to baseline was 1°C/s, while for thermal pain thresholds this ramp was chosen at 5°C/s. The baseline temperature was 32°C. Cut-off temperatures were set at 0°C and 50°C, respectively [20,21]. A standardized verbal instruction was read to the participants and a training test was performed before the actual tests to make sure that the participants understood the thermal test program [20].

### Psychometrical and physiological measures

The perceived intensity of the conditioning stimuli was assessed using a numerical scale from “0 = no stimulus could be detected” to “100 = the strongest stimulus imaginable”. The intensity rating was measured three times for each test, 3 seconds after the stimulus and at the beginning and ending of the thermal test. The mean value was calculated for statistics analysis. To determine whether the conditioning stimuli induced affective changes, participants rated their emotional reactions using the Positive and Negative Affect Schedule (PANAS), which composes of a list of 20 adjectives used to describe 10 positive emotions (the global Positive Affect Score) and 10 negative emotions (the global Negative Affect Score) after each thermal sensory test [31,32]. Respondents were required to indicate the extent to which they felt the emotions included on the schedule “during the thermal test” on a five-point scale (where 1 = very slightly or not at all, to 5 = extremely) [31,32].

Heart rate (HR) and skin temperature of contralateral orofacial region were measured using a Patient Monitor (NPB-4000, US) and an infrared thermometer (THERMOFOCUS, 01500 serien, Italy) continuously. HR and skin temperature were recorded seven times in each test (Figure 1) and the mean was used for further analysis [28].

### Statistical analysis

All statistical calculations were performed using SPSS 17.0 software for windows (IBM, inc., USA). The assumption of normal distribution of all data was investigated by the Shapiro-Wilk method. Paired *t*-test was used to compare the difference between baselines and stimuli tests. Differences between genders were evaluated by unpaired *t*-test. The changes under conditioning stimuli compared to baseline tests were calculated as delta values: △value = value (stimuli) – value (baseline), which was used in results and figures as “relative changes”. Differences between delta values within one session (dose dependent effect) were analyzed using two-way repeated measures ANOVA with the stimulus intensity as within-participant factor and gender as between-participant factor. To evaluate the multisensory effect, the delta values (“changes”) in multisensory stimulation test, gustatory test, 9000 lux light visual test, and 90 dB noise auditory test were compared using two-way repeated ANOVA with conditioning stimuli as within-participant factor, gender as between-participant factor. Post-hoc comparisons were estimated using Bonferroni post-hoc test with correction for multiple comparisons. *P* <0.05 was taken as an indication of a statistically significant difference.

## Results

### Participants

There was no significant age difference between female (25.4 ± 2.9 years, mean ± SD) and male (27.7 ± 3.2 years) participants (unpaired *t*-test age: *P* = 0.110).

### Psychometrical measures

**Figure 2:**
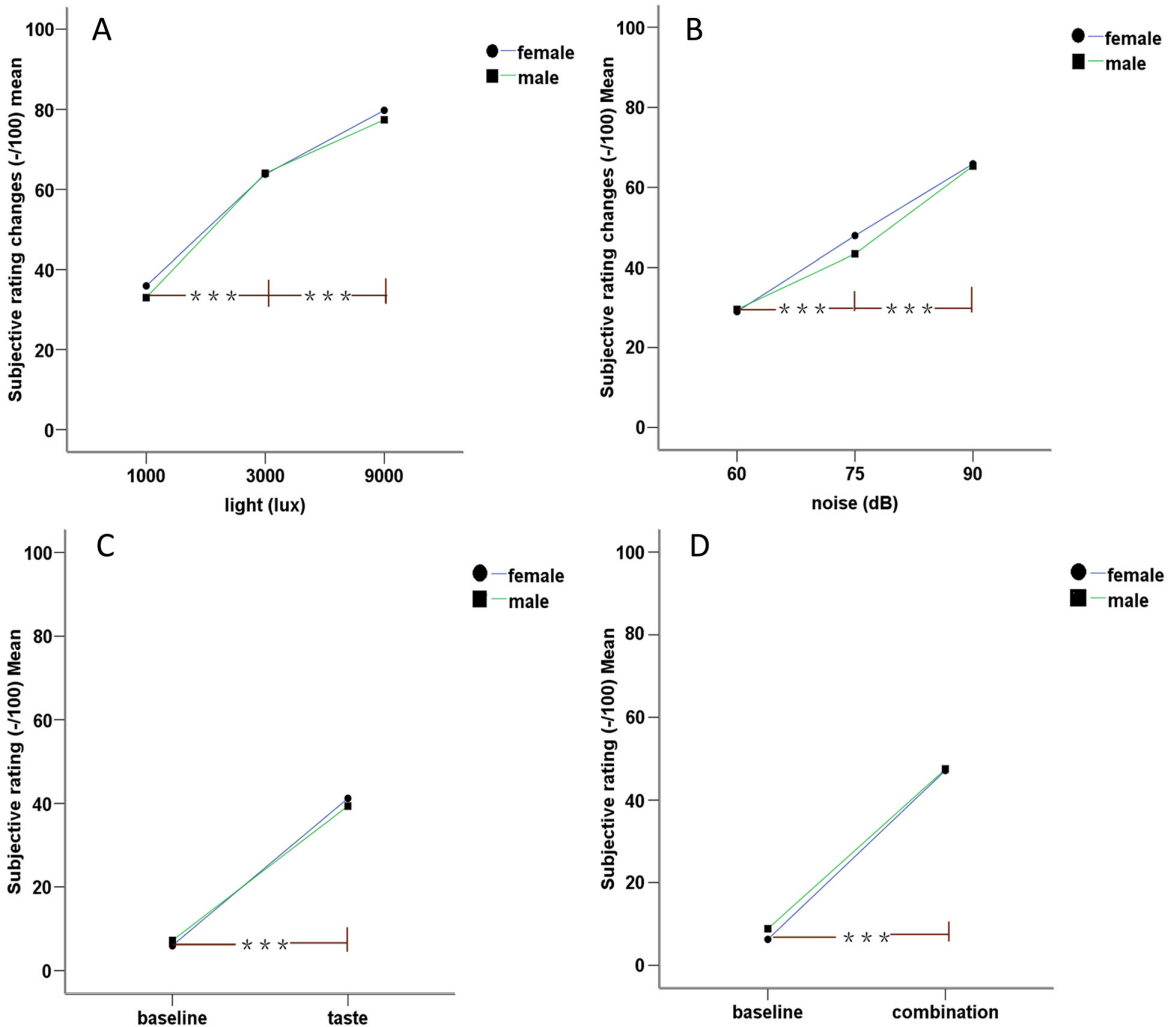
**Subjective rating (changes) to different conditioning stimuli.** The differences between test with conditioning stimuli and corresponding baseline were calculated as delta values: △value = value (conditioned) – value (baseline), which was used in figures (**A** visual and **B** auditory test) as “relative changes”. Compared with baseline, subjective stimulus intensity rating during gustatory and multisensory tests was significantly higher. The participants’ subjective rating changes were higher when they were exposed to stronger stimuli in visual and auditory tests. **(C)** gustatory test, **(D)** multisensory test. ***= *P* <0.001.

All the subjective intensity rating scores to different conditioning stimuli were normally distributed (Shapiro-Wilk, *P* ≥0.066). Compared with baseline, subjective stimulus intensity rating during conditioning stimuli tests was significantly higher in visual (paired *t*-test, *P* <0.001), auditory (*P* <0.001), gustatory (*P* <0.001), and multisensory test (*P* <0.001) (Table 1). The participants’ subjective rating differences between the condition with stimulus and corresponding baseline (“relative changes” in the text and figures) was higher when participants were exposed to stronger stimuli: significant intensity effect on subjective rating “relative changes”, *F* = 88.04, *P* <0.001 for visual test (Figure 2-A); *F* = 88.25, *P* <0.001 for auditory test (Figure 2-B). Post-hoc test for multiple contrasts indicated significant increases with increased intensity of light and noise (Figure 2-A,B). There were no gender differences in visual, auditory, gustatory, and multisensory tests (unpaired *t*-test, *t* ≤0.266, *P* >0.791).

**Table 1:** Subjective intensity rating to conditioning stimuli (−/100)

**Conditions**	**Baseline (mean ± SD)**	**With stimuli (mean ± SD)**	***P***
Visual	0 ± 0	59.0 ± 24.1	< 0.001
Auditory	18.8 ± 15.0	65.6 ± 24.1	< 0.001
Gustatory	6.6 ± 4.4	40.3 ± 17.3	< 0.001
Multisensory	7.5 ± 4.9	47.3 ± 22.8	< 0.001

Subjective rating to conditioning stimuli was assessed by numerical scale, in which “0 = no stimulus could be detected” to “100 = the strongest stimulus imaginable”. The comparisons between the conditions with stimuli and corresponding baseline were investigated by paired *t*-test.

**Figure 3:**
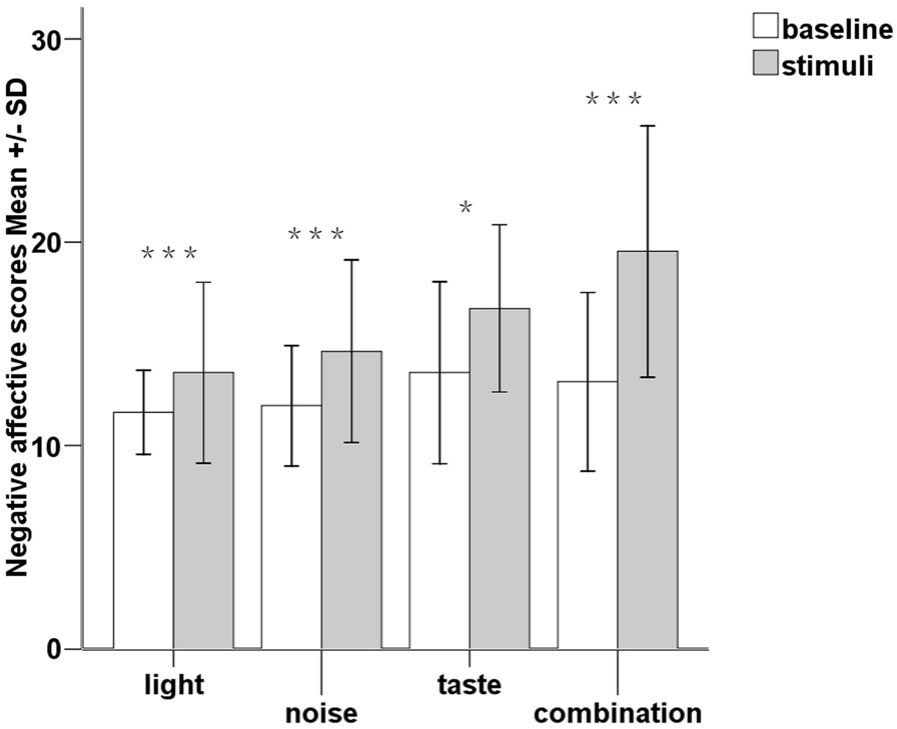
**Negative affective state differences between tests with conditioning stimuli (grizzle bars) and corresponding baselines (white bars).** The results are presented as means ± SDs of the negative affective scores form Positive and Negative Affect Schedule. The conditioning stimuli induced statistically significantly higher negative affective states compared with baseline. ***= *P* <0.001, * = *P* <0.05.

**Figure 4:**
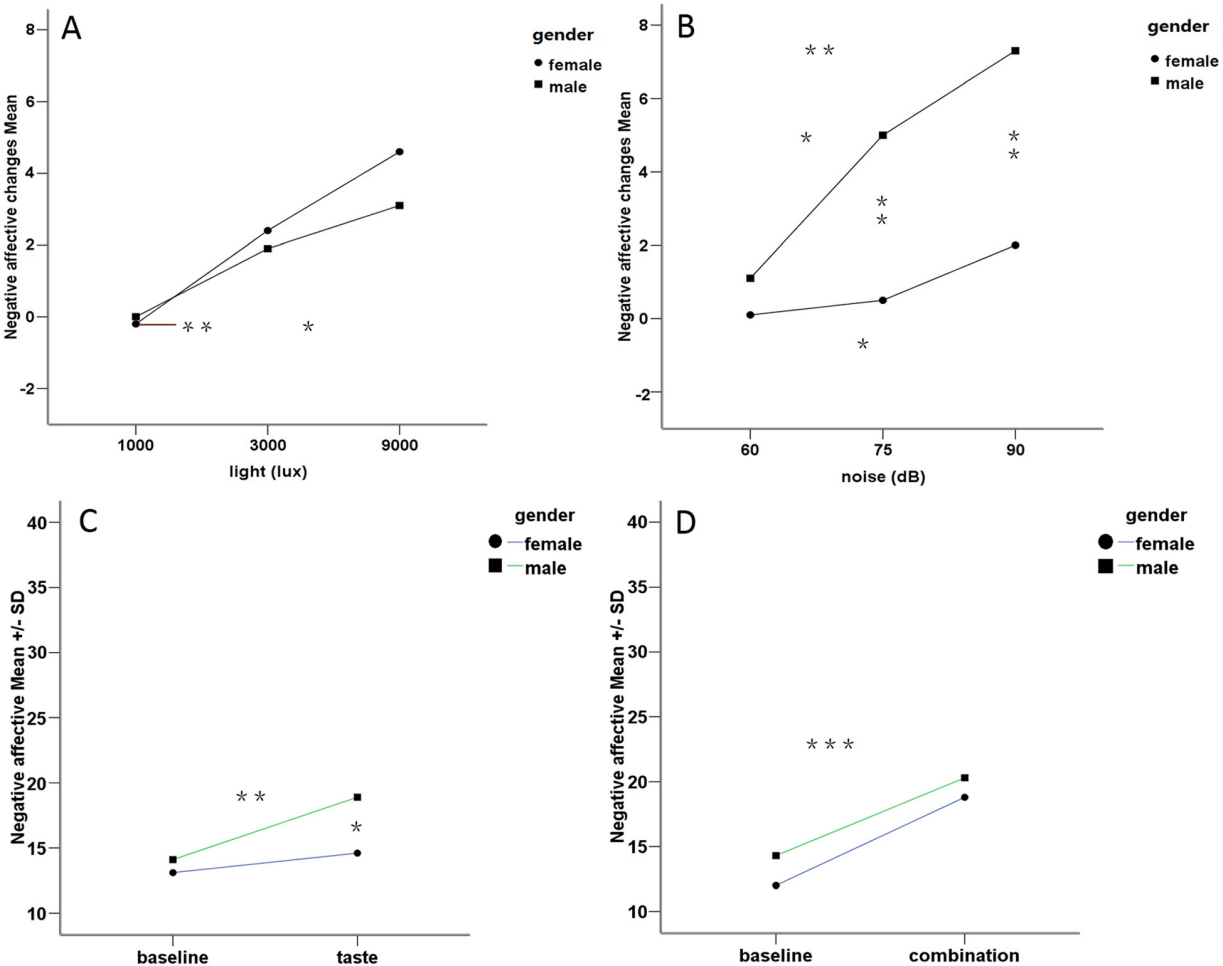
**Effects of conditioning stimuli on negative affective (changes) between genders.** The negative affective score differences between the condition with stimuli and corresponding baseline (as “changes” in the figure) were higher when the participants were exposed to more intense stimuli in visual **(A)** and auditory **(B)** tests. Besides, there were statistically significant gender differences in auditory test **(B)** and gustatory test **(C). (D)** indicates multisensory test. ***= *P* <0.001, **= *P* <0.01, *= *P* <0.05.

Most of the affective scores to different conditioning stimuli were normally distributed (Shapiro-Wilk, *P* >0.05). The conditioning stimuli induced statistically significantly higher negative affective states compared with baseline: visual test (baseline: 11.6 ± 2.1, mean ± SD, stimulus: 13.6 ± 4.5; paired *t*-test, *t* = −3.742, *P* <0.001); auditory test (baseline: 12.0 ± 3.0, stimulus: 14.6 ± 4.5; *t* = −5.182, *P* <0.001); gustatory test (baseline: 13.6 ± 4.5, stimulus: 16.8 ± 4.1; *t* = −2.656, *P* =0.016); multisensory test (baseline: 13.2 ± 4.4, stimulus: 19.6 ± 6.2; *t* = −6.719, *P* <0.001) (Figure 3). The negative affective score differences between the condition with stimuli and corresponding baseline (as “delta value” or “changes” in the text and figures) were higher when the participants were exposed to more intense stimuli: significant intensity effects on negative affective scores for visual test (*F* = 7.484, *P* =0.006, Figure 4-A), auditory test (*F* = 11.335, *P* <0.001, Figure 4-B). The Post-hoc test for multiple comparisons indicated a statistically significant increase in negative affective scores with increasing intensities in the light and noise tests (Figure 4-A,B). Besides, there were statistically significant gender differences, with males having higher negative affective scores when exposed to the same stimuli compared to females: in auditory test (unpaired *t*-test gender differences, *t* = −3.308, *P* = 0.004 for 75 dB noise; *t* = −3.519, *P* =0.002 for 90 dB noise, Figure 4-B) and gustatory test (*t* = −2.694, *P* = 0.015, Figure 4-C). As could be expected, all the stimuli failed to induce positive affective changes of the participants compared with baseline: visual test (paired *t*-test, *P* = 0.117), auditory test (*P* = 0.091), gustatory test (*P* = 0.169), and multisensory test (*P* = 0.080).

### Physiological measures

The heart rate and skin temperature data for different conditioning stimuli were normally distributed (Shapiro-Wilk, *P* ≥0.069). The conditioning stimuli did not change the two indices of physical arousal, heart rate and skin temperature, in comparison with the corresponding baseline values: pared *t*-test *P* value for differences (heart rate, temperature) in visual test (0.408, 0.162), auditory test (0.059, 0.574), gustatory test (0.195, 0.639) and multisensory test (0.137, 0.057).

### Thermal somatosensory sensitivity

**Figure 5:**
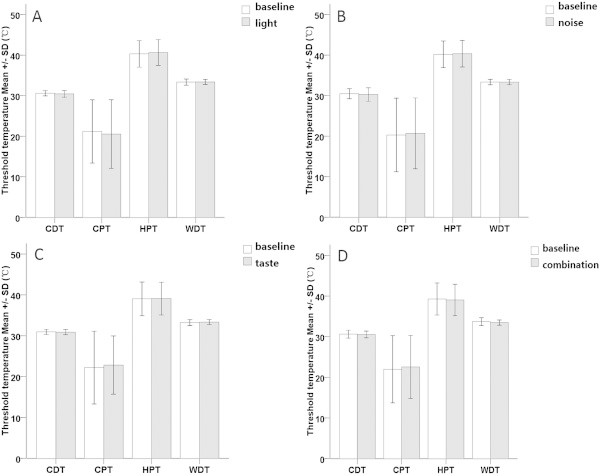
**Thermal sensitivity differences between tests with conditioning stimuli (grizzle bars) and corresponding baselines (white bars).** The results are presented as means ± SDs of the threshold values from visual test **(A)**, auditory test **(B)**, gustatory test **(C)** and multisensory test **(D)**. Compared with baseline (white bars), the sensitivity to thermal stimulus did not change when the participants were exposed to conditioning stimuli (grizzle bars). CDT = cold detection threshold, WDT = warmth detection threshold, CPT = cold pain threshold, HPT = heat pain threshold.

Most of the QST data in different conditioning stimuli were normally distributed only after logarithmic transformation (Shapiro-Wilk, *P* >0.05). Compared with baseline, the sensitivity to thermal stimuli did not change when the participants were exposed to conditioning stimuli: visual test (paired *t*-test *P* value for CDT, WDT, CPT, HPT were 0.074, 0.641, 0.165, 0.095 respectively; Figure 5-A), auditory test (paired *t*-test *P* value for CDT, WDT, CPT, HPT were 0.055, 0.922, 0.515, 0.418 respectively; Figure 5-B), gustatory test (paired *t*-test *P* value for CDT, WDT, CPT, HPT were 0.347, 0.207, 0.202, 0.729 respectively; Figure 5-C), multisensory stimulation (paired *t*-test *P* value for CDT, WDT, CPT, HPT were 0.526, 0.051, 0.737, 0.331 respectively; Figure 5-D).

### Multisensory stimulation effect

**Figure 6:**
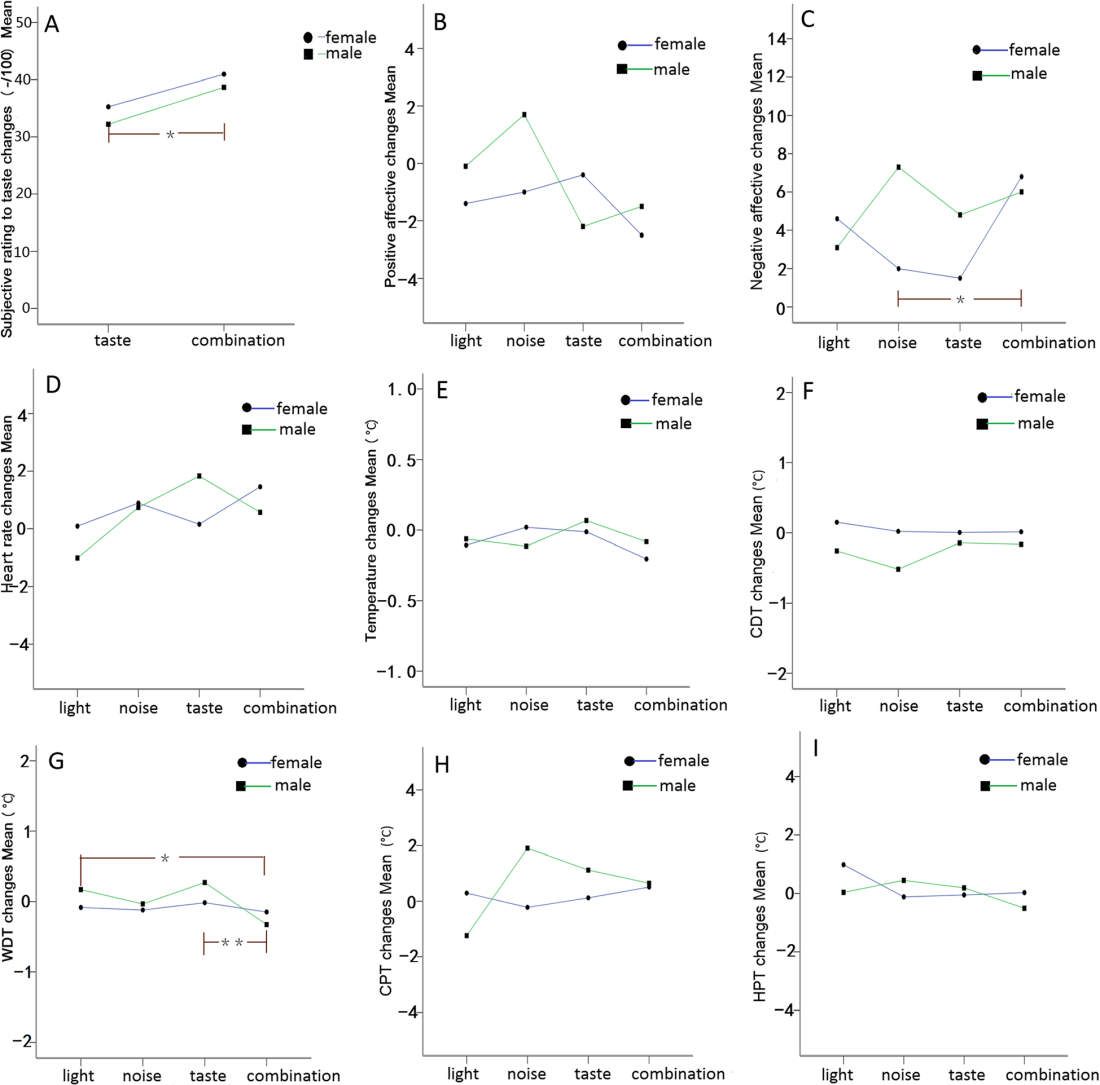
**Multisensory effects between genders.** To diminish the effect of timing, the delta values between test with stimuli and corresponding baseline, defined as “changes” in the figure was used. Multisensory conditioning stimulus led to a strengthened effect compared with single conditioning stimuli for several outcome parameters, i.e. subjective ratings to gustatory stimulus **(A)**, negative affective scores **(C)** and warmth detection threshold **(G)**. CDT = cold detection threshold, WDT = warmth detection threshold, CPT = cold pain threshold, HPT = heat pain threshold. Positive affective changes **(B)**, heat rate changes **(D)**, skin temperature changes **(E)**, cold detection threshold changes **(F)**, cold pain threshold changes **(H)**, and heat pain threshold changes **(I)** in different tests were also presented. **= *P* <0.01, *= *P* <0.05.

When the participants were exposed to multisensory (combined) conditioning stimuli, they subjectively rated higher “delta values” to the same bitter taste compared with in the condition of taste stimulus alone (whole group: *F* = 5.017, *P* = 0.038; male: *F* = 8.980, *P* = 0.015; female: *F* = 1.304, *P* >0.05 Figure 6-A). The female participants presented higher “delta values” for the negative affective scores during the multisensory stimuli condition than during noise stimulation alone (noise: 2.0 ± 2.4, mean ± SD; taste: 1.5 ± 6.3; combination: 6.8 ± 5.8; Post-hoc test between combination test and noise test, *P* = 0.011, Figure 6-C). The participants presented a statistically significant decrease in WDT (“delta” WDT decreasing) during multisensory conditioning stimuli compared with light stimulus alone (male: Post-hoc test, *P* = 0.019, Figure 6-G, Table 2) or taste alone (whole group: *P* =0.004; male: *P* = 0.003; female: *P* = 0.438, Figure 6-G, Table 2).

**Table 2:** Impact of gender on warmth detection threshold (WDT, °C) changes with different stimuli

**Gender**		**Visual (1)**	**Auditory (2)**	**Gustatory (3)**	**Multisensory (4)**	***F***	***P***	**1 vs .4**	**2 vs .4**	**3 vs .4**
Female	Mean	−0.08	−0.12	−0.02	−0.15	0.098	N.S	0.784	0.938	0.438
	SD	0.76	0.64	0.35	0.60					
Male	Mean	0.17	−0.03	0.27	−0.33	6.036	*0.003*	*0.019*	N.S	*0.003*
	SD	0.29	0.21	0.50	0.33					
Sum	Mean	0.04	−0.08	0.13	−0.24	2.241	N.S	0.058	0.387	*0.004*
	SD	0.57	0.46	0.45	0.48					

To evaluate the multisensory effect, the delta values (“changes”) in 9000 lux light visual test, 90 dB noise auditory test, gustatory test, and multisensory stimulation test were compared using two-way repeated ANOVA with conditioning stimuli as within-participant factor, gender as between-participant factor. The male participants presented a statistically significant decrease in “delta” WDT during multisensory conditioning stimuli compared with light stimulus alone (*P* = 0.019) or taste alone (*P* = 0.003). Italics values indicate statistically significant *P* values (*P* < 0.05).

## Discussions

This study investigated for the first time the effects of three unpleasant conditioning stimuli (bright light, chainsaw noise and bitter taste) on negative affective state, physiological arousal and skin thermal sensitivity. The three conditioning stimuli, as expected, all induced statistically significant increases in negative affective scores compared with baseline. Additionally, a dose dependent phenomenon was demonstrated in the visual and auditory tests (only one intensity gustatory stimulus was applied in this study). There were no significant changes in positive affective scores, physiological arousal (heart rate or skin temperature) between tests with and without conditioning stimuli. The skin thermal sensitivity (via, CDT, WDT, CPT, HPT) in the perioral region did not change significantly during conditioning stimuli tests compared with baseline. Combining the individual conditioning stimuli into a multisensory conditioning stimulus led to a strengthened effect compared with single conditioning stimuli for several outcome parameters, i.e. subjective ratings, negative affective scores and warmth detection threshold (WDT).

### Influence of conditioning stimuli on affective state

The experimental visual stimuli used in previous studies to examine the affective processes were composed of emotional videos [33,34] or photos [35,36]. Viewing faces with sad or happy expressions specifically evoked the expressed feelings in the viewer, defined as “emotional contagion” [35]. This emotional contagion was considered as a “prewired” decoding instrument, which was considered a fast, repeatable and stable process [35]. These visual stimuli activate an emotion perception process characterized by focusing attention to external events, and accurately understanding the information conducted, which need the full co-operation of the investigated individuals [11]. Additionally, some studies concluded that the experimentally induced changes in the affective state were obtained only after repeated stimuli presentation accompanied with specified verbal inductions [37]. Furthermore, in another Electroencephalography (EEG) study, emotional reactions could not be evoked by presenting emotional pictures alone [11]. In migraineurs, bright light often exacerbate the headache, which could be accounted for a pathophysiological hypothesis of a disturbance of the subcortical sensory modulation system [1,8]. Although the trigeminal autonomic reflex acts as a feed-forward system to facilitate the acute attack, the fundamental problem was thought to be in the brain [1]. The possible effect of bright light to experimentally evoke an affective state and somatosensory sensitivity changes has not been assessed before. The hypothesis of the present study was that bright light could evoke a negative affective state and change the somatosensory sensitivity. In the present experimental study, bright light successfully evoked a negative affective state in a dose-dependent manner according to the intensity of the light (Figure 4-A). Bright light may be a simple and effecient way to induce a negative affective state in human studies. However, bright light did not significantly change somatosensory sensitivity to thermal stimuli. This indicates that the light regulation of thermal somatosensory sensitivity may not exist in healthy humans without prior sensitization or predisposition.

Several studies have demonstrated that music is a powerful mediator of changes in affective state [38,39]. Functional neuro-imaging and lesion studies have shown that music-evoked affective state changes can modulate activity in virtually all limbic and paralimbic brain structures, such as amygdala, parahippocampal gyrus and hippocampus [12,40-42]. These structures are crucially involved in the initiation, generation, detection, maintenance, regulation and termination of changes in affective state [13]. Music may affect trained musicians and non-musicians differently and different individuals have different musical preferences and experiences, which may be important in studies of influence of music on different outcome parameters [13]. In contrast, to most people a chainsaw noise as used in the present study can be considered an unpleasant auditory stimulus. The participants in this study rated moderate to strong unpleasantness to the noise (Figure 2-B). The hypothesis of the present study was that an unpleasant auditory stimulus could increase negative affect and change somatosensoty sensitivity. The chainsaw noise successfully evoked a negative affective state in a dose-dependent manner as the intensity of the auditory stimulus increased (Figure 4-B). Again, chainsaw noise may be a simple and effective way to induce a negative affective state in human studies. However, the chainsaw noise did not significantly change somatosensory sensitivity to thermal stimuli. This indicates that auditory stimulus regulation of thermal somatosensory is not significant in healthy volunteers. However, in some pathological conditions, such as tension-type and cervicogenic headache, phonophobia is a common symptom [43]. Again, this may require a sensitized or predisposed somatosensory system.

It has been reported that sucrose reduces pain during the Cold Pressor Test (CPT) in 5- to 10-year-old children, and this kind of analgesia is dependent on the sweet taste preference [14]. However, sucrose was not an effective analgesia in adult women in the same study. The age-related decline in the analgesic efficacy of sucrose mirrors the age-related decline in the hedonic value of sweet tastes [44]. In a recent study, the taste of quinine gelatin (0.1%) failed to produce robust affective state changes [45]. However, in the present study the same stimulus evoked a significant change in negative affective state. These contradictory results may possibly been explained by the “preference theory”, since the subjective rating to the bitter taste in this study (40.3 ± 17.3, mean ± SD, in a 0–100 numerical scale) was much higher than the study by Horjales-Aaujo (29.7 ± 19.7) [14,45]. Despite the negative affective state, there were no changes in somatosensory sensitivity in healthy volunteers.

### Multisensory conditioning stimulus effects

There were several statistically significant effects of the multisensory conditioning stimulus, which indicated that the multisensory stimulation enhanced the effect of individual stimuli with regard to subjective rating changes, affective score changes and induced slight thermal sensitivity changes. This is in line with a neurophysiological study, which demonstrates a strong emotion enhancement effect by simultaneous presentation of congruent emotional pictures and music, regarding subjective ratings, peripheral and central physiological measures [11]. The findings also fit with a study, which shows that when either positive (joyful) or negative (fearful) music is played simultaneously with an emotionally neutral film clip, it evokes stronger signal changes in the amygdala and in areas of the ventrolateral frontal cortex, compared with when only music or only film clips are presented [46]. The activation increase may be due to the cross modal integration of three sensory stimuli, and this stronger activation possibly suggests enhanced activation in a distributed neuronal network, which needs to be confirmed in functional imaging studies [6].

### Physiological arousal changes

It has been proposed that most affective changes are associated with undifferentiated physiological activity changes [47]. Thus it seems reasonable to examine physiological arousal measurements related to affective reactions. Skin temperature and heart rate are the most frequently used physiological expressions of affective state [47]. However, in the present study, these two parameters did not change significantly in response to changes in negative affective state by the different conditioning stimuli. This is in line with another study, where it was demonstrated that the verbally reported affective experiences are not always consistent with the physiological parameters, especially for unpleasant stimuli [47]. On the other hand, it may be because of the limited number of participants or that the applied stimulus was inadequate for induction of a significant physiological change in arousal.

### Interactions between somatosensory sensitivity and conditioning stimuli

The perception of migraine headache, which is mediated by nociceptive signals, is exacerbated by exposure to environment stimuli, i.e. light or noise, which are mediated by optical and acoustic signals. Prolonged neuronal activation during a migraine attack is thought to induce peripheral and central sensitization along the trigeminovascular pathway, which stands to explain the throbbing headache [48], accompanying scalp and neck-muscle tenderness [49], and whole-body cutaneous allodynia [50]. Several theories exist, which predict the relationship between conditioning stimuli and somatosensory sensitivity. To develop a more comprehensive model of somatosensory modulation by conditioning stimuli, it may be useful to consider a theory of “motivational priming” [9]. It is proposed that affective state processing evoked by stimuli could be viewed as two opposing motivational systems. One system is appetitive and produces approach behaviors, and the other system is aversive and promotes avoidance behaviors [9]. Considerable evidence indicates that prior activation of these systems modulates defensive behaviors, which facilitates somatosensory capability [51]. These defensive behaviors increase in magnitude when the aversive system is primed by a negative affective state, i.e. anticipation of shock [52]. Conversely, this defensive response is inhibited, when the appetitive system is primed by a positive affective state [53]. According to the motivational priming theory, participants exposed to negative affective loading, whose aversive systems are activated, should get their perception facilitated [9,22]. However, somatosensory sensitivity to thermal stimuli was not changed during induction of negative affective states in the present study. Another perspective on somatosensory and affective processing is provided by the four-region neurobiological model, which indicates a complex relationship between acute pain and simple emotion [54]. For instance, fear and pain sites overlap in the anterior midcingulate cortex (aMCC) and this region is involved in avoidance behaviors; posterior midcingulate cortex (pMCC) has no consistent emotion activations, yet has robust nociceptive responses; subgenual anterior cingulate cortex (sACC) activation during noxious stimulation of the skin and viscera in a person-specific manner; the cingulate cortex is not uniformly involved in emotion, and not all pain-activation sites are associated with affect or emotion [54].

## Conclusions

The three conditioning stimuli induced significant increases in subjective rating and negative effective scores accompanied with no physiological arousal changes. These stimuli did not, however, modulate skin thermal sensitivity. The multisensory conditioning stimuli induced stronger effects on subjective rating and negative affect than individual conditioning stimuli alone. The often observed trigger or exacerbation of migraine by visual, auditory or gustatory stimuli may require a predisposed or sensitized nervous system.

## Abbreviations

MSI: Multi-modal sensory integration; IAPS: Pictures of facial affect or the international affective picture system; QST: Quantitative sensory testing; CDT: Cold detection threshold; WDT: Warmth detection threshold; CPT: Cold pain threshold; HPT: Heat pain threshold; PANAS: Positive and negative affect schedule; HR: Heart rate; EEG: Electroencephalography; aMCC: Anterior MidCingulate cortex; pMCC: Posterior MidCingulate cortex; sACC: Subgenual anterior cingulate cortex.

## Competing interests

The authors declare that they have no competing interests.

## Authors’ contributions

YG recruited the participants, carried out the study, performed the statistical analysis, and drafted the manuscript. SP and BHL participated in the design of the study, statistical analysis, and revision of the draft. XQ and WK helped to draft and revise the manuscript. All authors read and approved the final manuscript.
